# Linseed Oil Supplementation of Lambs’ Diet in Early Life Leads to Persistent Changes in Rumen Microbiome Structure

**DOI:** 10.3389/fmicb.2017.01656

**Published:** 2017-08-29

**Authors:** Tamsin Lyons, Tommy Boland, Sean Storey, Evelyn Doyle

**Affiliations:** ^1^Environmental Microbiology Group, School of Biology and Environmental Science and Earth Institute, University College Dublin Belfield, Ireland; ^2^School of Agriculture and Food Sciences, University College Dublin Belfield, Ireland

**Keywords:** rumen microbiome, dietary intervention, early life, linseed oil

## Abstract

Diet has been shown to have a significant impact on microbial community composition in the rumen and could potentially be used to manipulate rumen microbiome structure to achieve specific outcomes. There is some evidence that a window may exist in early life, while the microbiome is being established, where manipulation through diet could lead to long-lasting results. The aim of this study was to test the hypothesis that dietary supplementation in early life will have an effect on rumen microbial composition that will persist even once supplementation is ceased. Twenty-seven new-born lambs were allocated to one of three dietary treatments; a control group receiving standard lamb meal, a group receiving lamb meal supplemented with 40 g kg^-1^ DM of linseed oil and a group receiving the supplement pre-weaning and standard lamb meal post-weaning. The supplement had no effect on average daily feed intake or average daily weight gain of lambs. Bacterial and archaeal community composition was significantly (*p* = 0.033 and 0.005, respectively) different in lambs fed linseed oil throughout the study compared to lambs on the control diet. *Succinivibrionaceae*, succinate producers, and *Veillonellaceae*, propionate producers, were in a higher relative abundance in the lambs fed linseed oil while *Ruminococcaceae*, a family linked with high CH_4_ emitters, were in a higher relative abundance in the control group. The relative abundance of *Methanobrevibacter* was reduced in the lambs receiving linseed compared to those that didn’t. In contrast, the relative abundance of *Methanosphaera* was significantly higher in the animals receiving the supplement compared to animals receiving no supplement (40.82 and 26.67%, respectively). Furthermore, lambs fed linseed oil only in the pre-weaning period had a bacterial community composition significantly (*p* = 0.015) different to that of the control group, though archaeal diversity and community structure did not differ. Again, *Succinivibrionaceae* and *Veillonellaceae* were in a higher relative abundance in the group fed linseed oil pre-weaning while *Ruminococcaceae* were in a higher relative abundance in the control group. This study shows that lambs fed the dietary supplement short-term had a rumen microbiome that remained altered even after supplementation had ceased.

## Introduction

Ruminant livestock, such as cattle and sheep, rely wholly on microorganisms in their rumen to degrade fibrous feed and allow them to obtain nutrients from food. The rumen microbiome consists of billions of interacting species of bacteria, protozoa, archaea, fungi and bacteriophage working together to digest fiber, starch, fats and sugars, and produce volatile fatty acids (VFAs), primarily acetate, butyrate and propionate (Hobson and Stewart, 1997). Additionally, microorganisms act as a source of protein and B-vitamins for their host. In exchange, the animal provides a warm, stable and moist environment with a steady supply of nutrients, allowing microbes to thrive (Nocek and Russell, 1988; Hobson and Stewart, 1997; Russell and Rychlik, 2001; Chaucheyras-Durand and Ossa, 2014).

As a by-product of feed digestion, rumen microbes also produce compounds which cannot be used by the host, such as carbon dioxide and hydrogen. If hydrogen accumulates in the rumen it can be dangerous to the animal’s health and lead to bloating and decreased feed fermentation. Methanogens, a group of archaea, combat this problem by reducing hydrogen to methane (CH_4_) which is then removed from the body through eructation (Hobson and Stewart, 1997). This results in increased methane in the atmosphere and an energy loss of 2–13% for the animal, impacting feed efficiency in animal production (Johnson and Johnson, 1995).

Over the last decade, microbial community structure in the forestomach of ruminant animals has been increasingly linked with trends in feed efficiency, greenhouse gas (GHG) emissions and host health. It has become clear that certain rumen microbial profiles favor increased feed efficiency and decreased GHG emissions (Wanapat et al., 2015). A range of approaches such as increasing feed quality, increasing the ratio of concentrate to forage feed, using rumen modifiers such as defaunation or methanogenesis inhibitors (e.g., mevastatin), using production-enhancing agents such as ionophores and using lipids or fatty acids as dietary supplements have been investigated as a means of manipulating the rumen microbiome to achieve specific outcomes (Boadi et al., 2004; Knapp et al., 2014; Caro et al., 2016). Although these strategies have yielded varying levels of success, diet has been repeatedly shown to strongly influence rumen microbial structure (de Menezes et al., 2011; Ellison et al., 2014; Cobellis et al., 2016). For example, Petri et al. (2013) reported significant differences in rumen bacterial community structure in mature cattle fed a forage diet versus those on a high concentrate diet while Kocherginskaya et al. (2001) described differences in bacterial community composition in cattle fed hay diets compared to those fed corn diets.

Supplementation of ruminant diets with polyunsaturated fatty acids (PUFA) has been reported to reduce enteric methanogenesis (Martin et al., 2008; Petrie et al., 2009; Guyader et al., 2015). Soya oil and linseed oil, both plant based dietary supplements rich in PUFAs (n-6 acids and n-3 acids, respectively), reduced enteric methane emissions with no effect on milk production when introduced to the diet of cattle (Rowntree et al., 2010). Lillis et al. (2011) reported significant differences in bacterial community composition and methanogen abundance in the rumen of cattle receiving soya oil compared to those who were not. Furthermore, cattle fed soya oil had reduced fungal species richness in their rumens (Boots et al., 2013). These studies have stimulated interest in using diet to modify rumen microbiome structures to facilitate specific outcomes such as reduced GHG emissions, increased feed efficiency, etc. However, dietary supplements are expensive and long-term use may not be economically feasible or commercially practical.

Additionally, once established the mature rumen microbiome is quite stable and resilient and at present dietary interventions have only been reported to result in inconsistent or short-term changes in rumen microbial structure (Yáñez-Ruiz et al., 2015). Many interventions to date, such as antibiotic application and probiotic supplementation, have not been successful, with the microbiome composition and fermentation profile returning to the pre-treatment composition once treatment ceases (Weimer et al., 2010). In fact, Weimer et al. (2010) showed that even when the entire contents of the rumen were manually removed from one cow and swapped with rumen digesta sourced from a cow with a different rumen microbial profile, the rumen microbiome did not remain altered. Although the profile resembled that of the donor immediately after transfer, by the next sampling date (62 days later) the rumen microbial profile resembled the original.

Although the rumen is not functional immediately after birth, colonization begins straight away with bacterial communities reported to reach some degree of stability after 3 to 4 weeks (Abecia et al., 2014a; Rey et al., 2014; Guzman et al., 2015). This suggests that the period after birth could be critical for shaping the rumen microbiome to obtain long-lasting, stable results. Abecia et al. (2014b) fed kids and does a diet supplemented with bromochloromethane (BMC) for the first 3 months of life and the first 2 months’ post-partum, respectively, and reported a persistent alteration in the ruminal microbiome of kids 3 months after dietary intervention had ceased. BMC was used due to its ability to reduce enteric methane production in ruminants, however, commercial use of BMC was banned by the European Union in June 2000 (Regulation, 2000) so while this treatment is clearly effective at reliably altering the rumen microbiome, an alternative, less toxic compound is needed.

The aim of this study was to test the hypothesis that dietary supplementation in early life will have a significant effect on rumen bacterial and archaeal community composition that will persist even when supplementation is ceased. The supplement used in this study was linseed oil as it is non-toxic and has been previously shown to reduce enteric methane emissions by up to 64% (Martin et al., 2008; Rowntree et al., 2010) and thus may have potential in reducing GHGs from agriculture. New-born lambs were separated into three treatment groups based on diet. One group of lambs were fed a standard lamb meal (control group), a second group of lambs received a dietary supplement of linseed oil pre-weaning only (4 weeks) and were then placed on the control diet (L-P group) and a third group (L group) received the linseed oil supplement pre and post-weaning (∼14 weeks). At 16 weeks, the lambs were slaughtered and rumen microbial communities of all lambs were analyzed to determine if the linseed oil supplement had an effect and if this effect persisted once dietary supplementation had ceased.

## Materials and Methods

### Experimental Design

All procedures described were conducted under experimental license from the Irish Department of Health in accordance with the Cruelty to Animals Act 1876 and the European Communities (Amendments of the Cruelty to Animals Act 1876) Regulations, 1994. Twenty triplet-bearing ewes from the sheep herd at University College Dublin, Lyons Research Farm, Newcastle, Co., Dublin, Ireland were used in the current study. All ewes were bred using artificial insemination and lambed within 1 day of each other.

At 24 h post-partum, 20 triplet bearing and rearing ewes were enrolled in the study. Ewes were blocked based on combined litter weight (11.6 ± 1.51kg) and treatments were balanced for maternal live weight at parturition. The experimental design was a 2 × 2 factorial design. From birth to 6 weeks post-partum ewes (and lambs) were offered a grass silage based diet plus one of two concentrate types (with linseed oil (L) or without linseed oil (C) inclusion). After weaning lambs were offered one of the two concentrate types described above to meet 100% of their nutrient requirements. This lead to four post-weaning dietary treatments: C:concentrate without linseed oil fed both before and after weaning, L:concentrate containing linseed oil fed both before and after weaning, L-P:concentrate with linseed oil fed before weaning and concentrate without linseed oil fed post-weaning, L-POST: concentrate without linseed oil fed before weaning and concentrate containing linseed oil fed post-weaning.

The control diet contained 47.5% barley, 27% soybean meal, 10% citrus pulp, 9% soya hulls, 4% minerals and vitamins, and 2.5% cane molasses on a fresh weight basis. For the linseed diet barley inclusion level was reduced by 4% and replaced with 4% linseed oil. Ewes and lambs were housed in straw bedded pens and for the first 2 weeks of the trial, lambs had ad-lib access to the ewes’ milk. Ewes were fed 3 kg/head/day of concentrates which was gradually reduced to 1.5 kg/head/day over several weeks up to weaning. At 2 weeks of age, a creep area was created in the corner of each pen to which only lambs had access. Lambs were offered ad-lib creep meal concentrates according to the dietary treatment they were on.

Average feed intake of lambs was calculated each morning. Daily weigh back measurements of feed were taken and averaged among all trial lambs. Lambs were offered ad-lib concentrates and water. At approximately 6 weeks of age, when average lamb intake was above 550 g per day, lambs were weaned and ewes were removed from the pens. After weaning half the lambs on the control diet (C) were placed on the linseed diet (L) and vice versa. This resulted in the four treatment groups described- -three of which were analyzed in the current study (C, L and L-P). Each of these treatment groups consisted of 15 lambs, 9 of which were analyzed in this study, leading to a total of 27 lambs analyzed.

All groups were balanced for live weight (19 kg ± 3.2) and sex. Lambs were given a 7-day diet acclimation period. At 8 weeks of age, lambs were moved into individual pens where dry matter intake (DMI) was recorded. Lambs were offered ad-lib concentrates at approximately 110% of their DMI from the previous day. During the experimental period, live weight was recorded weekly using a calibrated Tru-Test SR 3000 electronic weighing scales (Tru-Test Ltd., Auckland, New Zealand). The weighing scales were calibrated each time with known weights.

Once they achieved a live weight of 41 kg (± 2.1kg), at approximately 111 (± 14) days, lambs were brought to Kildare Chilling Co. (Kildare Town, Co., Kildare, Ireland) for slaughter. The stomach was dissected and solid rumen digesta was removed, squeezed between two layers of cheesecloth and an 8 ml aliquot from the resulting collected rumen fluid was transferred into a labeled plastic vial containing 2 ml of trichloroacetic acid (50%). This sample was then frozen at -20°C for VFA and NH_3_ analysis. Unfiltered rumen solids were collected from 6 sites within the rumen, combined and stored at -80°C for microbial analysis.

### Volatile Fatty Acid Analysis

Rumen fluid samples were analyzed for VFA concentration by gas chromatography (Varian 3800, gas chromatograph) using a CP-wax 58, 25 m × 0.53 mm capillary column (Varian BC, The Netherlands) as previously described by Hart et al. (2009).

### DNA Extraction and Microbial Community Analysis

DNA was extracted from freeze-dried, unfiltered rumen solid samples from all 9 animals in each treatment group (27 samples in total) using the PCSA method described by Lueders et al. (2004), with phenol-chloroform added prior to the bead beating step, and stored at -20°C until needed. DNA concentration and purity were assessed using a Nanodrop^TM^ spectrophotometer (Thermo Scientific, Waltham, MA, United States).

Bacterial and archaeal community structures were analyzed using amplicon sequencing on an Illumina Miseq platform using the method described by Kozich et al. (2013). Briefly, once DNA was extracted and purified 1 μl target DNA was added to a well on a 96-well PCR plate already containing 17 μl of Accuprime Pfx Supermix (Invitrogen, Thermo Fisher Scientific, Dublin, Ireland) and 2 μl of a primer set targeting the V4 region of the 16S rRNA gene (Kozich et al., 2013). PCR conditions consisted of a hot start at 95°C for 2 min, followed by 95°C for 20 s, 55°C for 15 s and 72°C for 5 min (30 cycles) with a final elongation of 72°C for 10 min. PCR products were visualized on a 1.2% (w/v) agarose gel (Roche Diagnostic, Basel, Switzerland).

PCR products were then cleaned and normalized using the SequalPrep Normalization Plate Kit (Invitrogen, Thermofisher, Waltham, MA, United States) according to manufacturer’s instructions. DNA concentration was determined using a Qubit fluorometer and dsDNA kit (Thermofisher Scientific, Dublin, Ireland) to ensure a concentration ≥100 ng was present. The amplicon pool was then sent to the Centre for Genomic Research, University of Liverpool for sequencing on an Illumina Miseq platform. Sequence files associated with each sample have been submitted to the NCBI Sequence Read Archive (Accession no. PRJNA393972).

MiSeq sequencing data was initially processed using the mothur program developed by Schloss et al. (2009). Illumina adapter sequences were trimmed by cutadapt ver.2.1.1 using option -O 3. Sickle ver.1.2 was used to further trim the data with a quality score of ≥20. Reads <10 bp after trimming were removed. Each read was then trimmed to a maximum of 275 bp and ambiguous bases were removed. Sequences which contained homopolymer runs >8 bases were discarded. After trimming, identical sequences were grouped into ‘unique’ sequences. Chimeric sequences were identified using the UCHIME algorithm within mothur and were then removed. Sequences were assigned to operational taxonomic units (OTUs) using the ‘cluster’ command and the average neighbor algorithm. All subsequent OTU-based analyses were performed using a cutoff of 0.03. Taxonomy was assigned to all remaining aligned sequences by comparing processed data to the silva databases for bacteria and archaea independently^1^.

### Statistical Analysis

Group means were calculated for VFA concentrations, average daily feed intake and average daily gains within treatment groups. Analysis of variance (ANOVA) and *post hoc*, Tukey HSD tests were carried out within the R software environment using the ‘lsmeans’ package to analyze differences between means. Multivariate analysis was carried out using PRIMER-E v.7 software with the PERMANOVA add on. Similarity matrices were constructed for samples using Bray-Curtis similarities on standardized, fourth root transformed abundance data. Distance-based permutational multivariate analysis of variance (PERMANOVA; Anderson et al., 2001) was then performed to test the null hypothesis that there were no differences in microbial community structure across treatments at a significance level of α = 0.05 based on 9999 possible permutations. nMDS plots were constructed to visualize the data. Similarity percentages (SIMPER) were calculated using Bray-Curtis similarities to evaluate the level to which each family contributed to the difference in community structures between groups. Effects and differences were declared as significant if *p* ≤ 0.05 and trends if *p* ≤ 0.2.

## Results

At 2 weeks of age, twenty seven lambs were offered ad-lib access to either a standard lamb meal (control diet) or the standard lamb meal supplemented with 4% linseed oil (linseed diet). Once weaned (∼6 weeks of age), half of the lambs receiving the linseed diet (9 animals) were switched to the unsupplemented control diet, while the remainder (9 animals) continued on the linseed diet. Lambs on the control diet (9 animals) continued to receive the standard lamb meal throughout the whole study period. Supplementation of the standard lamb diet with linseed oil of (40 g kg^-1^ DM) had no significant effect on either average daily intake (*p* = 0.11) or average daily weight gain (*p* = 0.437) of the lambs (**Supplementary Figure S1**).

Lambs were slaughtered after 16 weeks and the effect of diet on rumen VFA profiles and bacterial and archaeal community structures examined. DNA was extracted from rumen solids from all animals and amplicon libraries were constructed based on the V4 region of the 16S rRNA gene and sequenced using the Illumina MiSeq platform. Following initial processing, an average of 52,672 good quality sequences were obtained, with sequence numbers per sample ranging from 3,043 to 148,437 (median of 36,260). A total of 265 unique OTUs that could be taxonomically classified to genus level were identified across all samples.

### Lamb Rumen Microbiome

Twenty-three distinct bacterial phyla were identified in the rumen of the control group of lambs that were fed standard lamb meal throughout the study. *Bacteroidetes* (39.43%) and *Firmicutes* (34.29%) dominated the rumen microbiome of these lambs 16 weeks after birth. The relative abundance of *Proteobacteria* was also high (16.64%) in the developing rumen (**Supplementary Table S1**).

**FIGURE 1:**
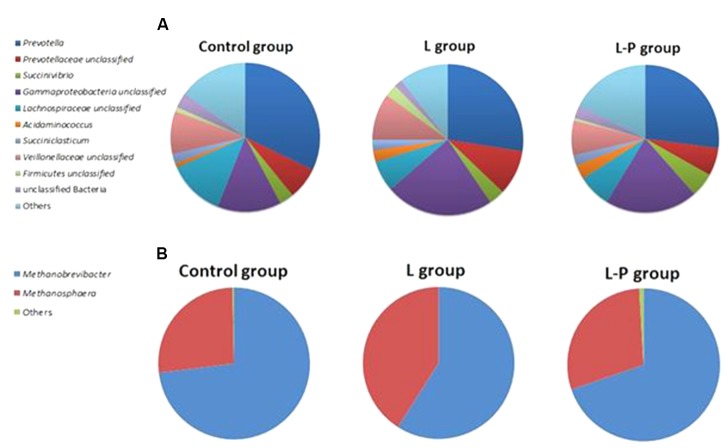
Relative abundance of **(A)** bacterial and **(B)** archaeal genera in the rumen of lambs fed standard lamb meal (Control group), lambs fed linseed oil for 16 weeks (L group) and lambs fed linseed oil pre-weaning only (L-P group).

Of the bacterial genera detected in the lamb rumen, the relative abundance of the *Prevotella* was highest, accounting for 31% of total bacterial sequences (**Figure 1A**), and a further 7% of bacterial sequences were associated with unclassified members of this family. Other bacterial genera that were present in relatively high abundance in the rumen of these lambs were unclassified genera from *Gammaproteobacteria* (13%), *Lachnospiraceae* (12%), and *Veillonellaceae* (9%). *Succinivibrio*, *Succiniclasticum* and unclassified genera of *Firmicutes* and *Clostridiales* were also present but their relative abundance accounted for less than 3% of all bacterial sequences. It should be noted that 3% of bacterial sequences detected in the rumen of the control lambs could not be assigned taxonomically at any level and 72 OTUs could only be assigned to family level.

Only seven genera of archaea were identified in the rumen of lambs in the control (no linseed supplementation) group. Of these, *Methanobrevibacter* was by far the most relatively abundant genus followed by *Methanosphaera* (**Figure 1B**). These two genera alone accounted for 99.35% of all archaeal sequences identified across all samples within this treatment group. The remaining sequences were assigned to *Methanosarcina*, *Methanomassiliicoccus*, unclassified *Thermoplasmata*, unclassified *Euryarchaeota* and unclassified *Nitrososphaera.*

### Effect of Linseed Oil Supplementation on Rumen Microbiome

**FIGURE 2:**
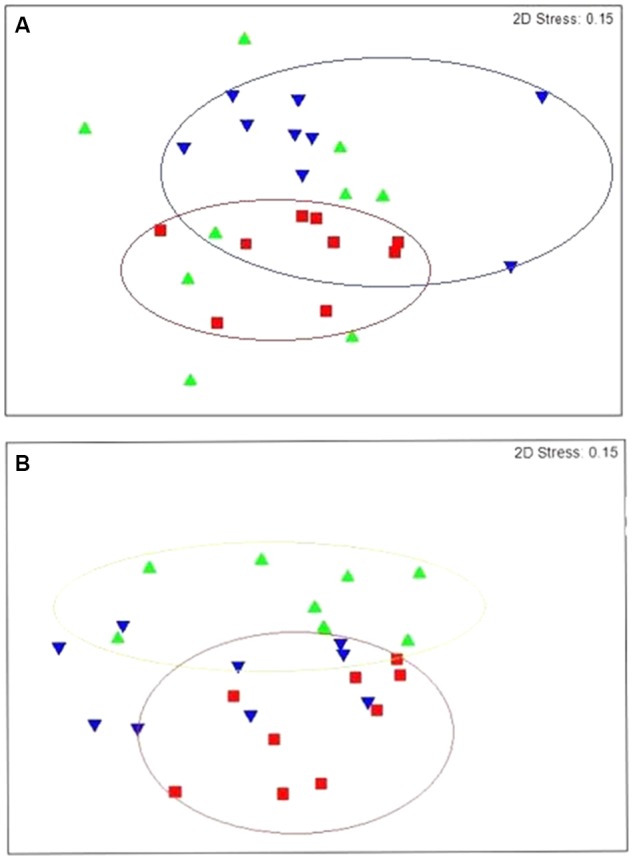
Non-metric multidimensional scaling (nMDS) plot of rumen **(A)** bacterial community structures and **(B)** archaeal community structures at genus level. Control lambs (C) are represented by the aaa symbol, lambs fed linseed oil for 16 weeks (L) are represented by the bbb symbol and lambs fed linseed oil pre-weaning only (L-P) are represented by the aaadown symbol.

*Prevotella* (27.48%), unclassified *Gammaproteobacteria* (23.16%), unclassified *Veillonellaceae* (9.97%) and unclassified *Lachnospiraceae* (6.57%) also dominated the rumen of lambs receiving the diet supplemented with linseed oil throughout the study (L) (**Figure 1A**). n-MDS ordination revealed that bacterial communities in these lambs (L) appeared to cluster together (**Figure 2A**) and PERMANOVA analysis confirmed there were significant (*p* = 0.033) differences in rumen bacterial community structure of these animals compared to the control animals.

Similarity percentage (SIMPER) analysis revealed that the main contributors to the dissimilarity in bacterial community structures between the lambs fed the linseed diet and those fed the control diet included *Succinivibrio*, unclassified *Gammaproteobacteriaceae, Selenomonas*, unclassified *Veillonellaceae, Dialister, Bifidobacterium*, unclassified *Prevotellaceae, Roseburia, Ruminococcus*, unclassified *Erysipelotrichaceae, Erysipelotrichaceae_incertae_sedis*, and unclassified *Firmicutes* (**Table 1**). Together these bacterial genera explained 21.16% of the dissimilarity in bacterial community structures.

**Table 1:** Similarity percentage (SIMPER) analysis of bacterial genera accounting for 21.16% of dissimilarity calculated between the ruminal community structures of groups C (control group) and L (group receiving linseed oil supplement throughout) after 16 weeks.

Genus	Contribution to community dissimilarity (%)	Relative abundance (%)	
		Control group	L group
*Succinivibrio*	2.54	0.95	1.15
*Gammaproteobacteriaceae* unclassified	2.19	1.82	2.06
*Selenomonas*	1.89	0.69	1.10
*Veillonellaceae* unclassified	1.82	1.66	1.73
*Dialister*	1.82	0.61	0.71
*Bifidobacterium*	1.66	0.06	0.41
*Prevotellaceae* unclassified	1.62	1.59	1.73
*Roseburia*	1.60	0.55	0.53
*Ruminococcus*	1.58	0.77	0.71
*Erysipelotrichaceae* unclassified	1.54	0.61	0.52
*Erysipelotrichaceae_incertae_sedis*	1.46	0.41	0.11
*Firmicutes* unclassified	1.45	0.93	0.99

Clear clustering of archaeal communities in the control lambs from the lambs on the linseed supplemented feed was also observed (**Figure 2B**) and PERMANOVA confirmed these communities were significantly different and this difference was most pronounced at genus level (*p* = 0.005). Although the same two genera of archaea, *Methanobrevibacter* and *Methanosphaera*, dominated the rumen of lambs receiving the supplement, the relative abundance of these genera differed significantly (*p* = 0.005) from the non-supplemented lambs. The relative abundance of *Methanobrevibacter* was 58.75% in the lambs receiving linseed compared to 73% in those that didn’t. In contrast, the relative abundance of *Methanosphaera* was significantly higher in the animals receiving the supplement compared to animals receiving no supplement (40.82 and 26.67%, respectively).

**FIGURE 3:**
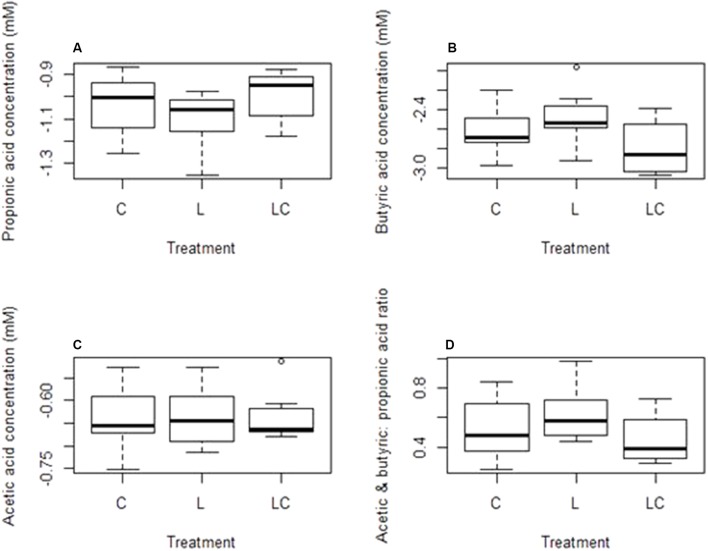
Concentration of volatile fatty acids (VFAs) measured after 16 weeks in the rumen of lambs on the control diet (C), lambs fed linseed oil for 16 weeks (L) and lambs fed linseed oil pre-weaning only (L-P)- **(A)** propionic acid, **(B)** butyric acid, **(C)** acetic acid, **(D)** ratio of acetic plus butyric acid to propionic acid.

No significant differences were observed in the concentrations of propionic, butyric or acetic acid produced by lambs fed the standard meal and those receiving the linseed oil supplementation (**Figure 3**).

### Persistent Effect of Early Life Dietary Supplementation

The rumen bacterial community structure of lambs that only received linseed oil during the pre-weaning stage (L-P) was also found to be significantly (*p* = 0.015) different from the control group (**Figure 2A**). SIMPER analysis revealed that 14 bacterial genera including *Succinivibrio, Bifidobacterium*, *Dialister, Lachnospiraceae_incertae_sedis* and unclassified *Gammaproteobacteria* were responsible for 21.28% of the dissimilarity (**Table 2**).

**Table 2:** Similarity percentage (SIMPER) analysis of bacterial families accounting for 21.28% of dissimilarity calculated between the ruminal community structures of groups C (control group) and L-P (group receiving linseed oil supplement pre-weaning only) after 16 weeks.

Genus	Contribution to community dissimilarity (%)	Relative abundance (%)	
		Control group	L-P group
*Succinivibrio*	2.35	0.95	1.46
*Bifidobacterium*	2.10	0.06	0.63
*Dialister*	1.83	0.61	1.01
*Lachnospiraceae_incertae_sedis*	1.62	0.26	0.67
*Gammaproteobacteria* unclassified	1.58	1.82	2.03
*Treponema*	1.50	0.66	0.57
*Veillonellaceae* unclassified	1.46	1.66	1.67
*Erysipelotrichaceae* unclassified	1.36	0.61	0.74
*Acidaminococcus*	1.33	0.96	1.32
*Roseburia*	1.29	0.55	0.50
*Prevotellaceae* unclassified	1.29	1.59	1.64
*Ruminococcus*	1.27	0.77	0.68
*Ruminococcaceae* unclassified	1.17	0.87	0.70
*Succiniclasticum*	1.13	1.20	1.10

Although there appeared to be increased archaeal species diversity in the group receiving linseed oil pre-weaning (L-P group) (**Table 3B**), community structure was not significantly (*p* = 0.12) different to that of the control group. There were no significant differences recorded in VFA concentrations between the L-P group and the control group (**Figure 3**).

**Table 3:** Species richness (d), species evenness (J′), and Shannon diversity [H′(loge)] of communities in the rumen of lambs on the control diet (Control), lambs fed linseed oil for 16 weeks (L group), and lambs fed linseed oil pre-weaning only (L-P group)- **(A)** bacterial communities, **(B)** archaeal communities.

Treatment	Species richness (d)	Species evenness (J′)	Shannon diversity index H′(loge)
**(A) Bacteria**			
Control	17.66081 (± 3.68)	0.948102 (± 0.007)	3.968839 (± 0.22)
L group	15.26875 (± 1.87)	0.947861 (± 0.005)	3.827596 (± 0.12)
L-P group	20.00073 (± 4.93)	0.948013 (± 0.014)	4.102832 (± 0.25)
**(B) Archaea**			
Control	0.72 (± 0.79)	0.4 (± 0.08)	0.42 (± 0.46)
L group	0.7 (± 0.5)	0.56 (± 0.09)	0.49 (± 0.33)
L-P group	0.99 (± 0.68)	0.61 (± 0.06)	0.65 (± 0.42)

### Differences in the Effect of Short-Term and Long-Term Dietary Supplementation

Comparison of lambs fed the linseed oil supplemented feed for ∼14 weeks (L group) and lambs fed linseed oil pre-weaning only (∼4 weeks; L-P group) revealed significant (*p* = 0.003) differences between their rumen bacterial communities. Clear clustering of bacterial communities in these two groups of lambs can be seen on the nMDS plot (**Figure 2A**) and bacterial species diversity was higher (*p* = 0.02) in the group that only received the linseed oil during the pre-weaning period compared to the group that received it throughout (**Table 3A**). Furthermore, some differences were detected in VFA concentrations between these two groups of lambs, with a trend toward increased propionate and decreased butyrate in the animals receiving the supplement during pre-weaning only (*p* = 0.16 and *p* = 0.083, respectively). However, no significant differences in archaeal diversity (**Table 3B**) or community structure (*p* = 0.99 and 0.29, respectively) were observed.

### Associations between Rumen Bacteria and VFAs

**FIGURE 4:**
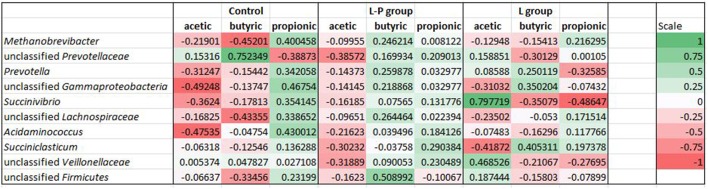
Heat maps showing correlations between the relative abundance of sequences assigned to each bacterial genus and VFA concentrations (acetic, butyric and propionic) in the rumen of lambs fed the control diet (C), lambs fed linseed oil pre-weaning only (L-P) and lambs fed linseed oil for 16 weeks (L). Pearson’s correlation coefficients (r) are given, with *r* < 0 indicating a negative correlation (red), *r* = 0 indicating no correlation (white) and *r* > 0 indicating a positive correlation (green).

Pearson’s correlation coefficient (r) was used to determine if any significant correlations could be observed between VFA profiles in the rumen of the lambs fed the different diets and any of the ten most relatively abundant bacterial genera (**Figure 4**). In lambs that received the unsupplemented diet throughout the study period (control), all the dominant genera except the unclassified *Prevotellaceae* were positively correlated with propionic acid. The unclassified *Prevotellaceae* were positively correlated with acetic and butyric acid (*r* = 0.15 and 0.75, respectively) while the other abundant genera had no correlation or were negatively correlated with these two VFAs.

A similar pattern was observed with the lambs fed the linseed oil supplemented diet in the pre-weaning period only (L-P group), with the relatively most abundant bacterial genera positively correlated with propionic acid with one exception, an unclassified *Firmicutes* (*r* = -0.1). However, in contrast to the control group, many of these genera were also positively correlated with butyric acid. Similar to the control group, most were negatively correlated with acetic acid.

A different profile was observed for the lambs fed the supplemented diet throughout the study period (L group). These lambs had less bacterial sequences positively correlated with propionic acid and negatively correlated with acetic acid. Although four of the most relatively abundant sequences were positively correlated with propionic acid, this correlation was weak. Relative abundances of *Prevotella, Succinivibrio* and an unclassified genus of *Veillonellaceae* were all negatively correlated with propionic acid concentrations (*r* = -0.33, -0.49, and -0.28, respectively). Relative abundances of *Succinivibrio*, and an unclassified *Veillonellaceae* were positively correlated with acetic acid (*r* = 0.79 and 0.47, respectively) while *Succiniclasticum, Prevotella* and an unclassified *Gammaproteobacteria* were positively correlated with butyric acid (*r* = 0.41, 0.25, and 0.35, respectively).

## Discussion

Addition of linseed oil to ruminant diets has been suggested as a potential means of reducing methane emissions (Machmüller et al., 1998; Rowntree et al., 2010). Methanogens in the rumen require hydrogen, synthesized by bacteria and protozoa during microbial fermentation of feed, to produce CH_4_. Therefore, it has been suggested that hydrogen is the limiting factor in CH_4_ production and reduced H_2_ availability could lead to reduced CH_4_ yield. Although the exact mechanism by which linseed oil supplementation suppresses CH_4_ production is not known, it has been reported that the supplement may reduce the amount of H_2_ available to methanogens in the rumen via a toxic effect on bacterial and protozoal H_2_ producers in the rumen (Morgavi et al., 2010) and during biohydrogenation of the polyunsaturated fatty acids in the oil (Czerkawski, 1986). To-date, however, any beneficial effects observed with adult animals have not persisted once dietary intervention has ceased (Yáñez-Ruiz et al., 2015). The current study examined the effect of linseed oil supplementation on the early life microbiome of lambs to determine if this dietary intervention exerted any effect on rumen microbial community structure during colonization of the rumen and if this effect varied depending on how long the intervention lasted. One of the concerns regarding any dietary supplement is whether it has an adverse effect on the animal. Linseed oil had no negative effect on the daily weight gain or average daily intake of lambs in the current study.

### Early Life Rumen Microbiome Structure of Lambs

Similar to other reports (Stevenson and Weimer, 2007; Lee et al., 2012; Henderson et al., 2015; Lopes et al., 2016), *Prevotella*, was the most relatively abundant genus in the rumen of lambs fed a standard lamb meal, accounting for almost one third of total bacterial sequences present. This genus is considered part of the core rumen bacterial community and important for organic matter degradation (Henderson et al., 2015). Based on the physiology of culturable members of this genus, *Prevotella* spp. are thought to be propionate and succinate producers (Bryant and Small, 1956; Strobel, 1992). Unclassified genera of *Gammaproteobacteria* also had high relative abundance in the lambs’ rumen; this finding was also reported by Wang et al. (2017). Unclassified *Lachnospiraceae*, a family reported to be positively correlated with gross feed efficiency (Jewell et al., 2015) were also present at high relative abundance. Similarly, *Veillonellaceae*, another family in relatively high abundance in lambs fed the unsupplemented diet, were identified as gain-associated bacteria in the rumen of steers (Myer et al., 2015), although Li and Guan (2017) reported that members of this family were associated with low feed efficiency. The *Veillonellaceae* family are known to produce propionate as a major fermentation product (Myer et al., 2015). *Prevotellaceae, Gammaproteobacteria, Lachnospiraceae*, and *Veillonellaceae* have also all been recently identified as part of the core rumen microbiome in cattle (Li and Guan, 2017).

The high number of unclassified sequences detected in this study has also been reported by other groups (Creevey et al., 2014; Liu et al., 2016) and is not surprising given the large percentage of rumen bacteria that still remain unculturable. The generation of a reference set of rumen microbial genome sequences by initiatives such as the Hungate 1000 should facilitate more accurate classification in the future (Creevey et al., 2014).

### Effect of Linseed Oil on Microbial Community Structure and VFA Profiles

Inclusion of linseed oil in the lambs’ diet for ∼14 weeks significantly changed ruminal bacterial and archaeal community structures. Similar results were observed by Lillis et al. (2011) when they analyzed the ruminal bacterial communities of adult cattle fed a diet supplemented with soya oil and those not. Soya oil, like linseed oil, contains polyunsaturated fatty acids and has also been shown to reduce enteric methane emissions when included in ruminant diets (Petrie et al., 2010). One of the top contributors to dissimilarity in bacterial communities between the control lambs and those fed linseed oil (L group) in the current study was the succinate producing (Russell and Rychlik, 2001) bacterial family, *Succinivibrionaceae*, which had a higher relative abundance in the L group. A previous study on tammar wallabies also reported high relative abundances of *Succinivibrionaceae* in the foreguts of wallabies and this was suggested to be an important contributing factor to the animals’ low enteric methane emissions (Pope et al., 2011). *Veillonellaceae*, known to be associated with propionate production (Reichardt et al., 2014; Lecomte et al., 2015; Myer et al., 2015), were present in a higher relative abundance in the L group while *Ruminococcaceae*, a family linked with higher CH_4_ emitters (Kittelmann et al., 2014), was present in a lower relative abundance in the L group compared with the control group. Higher relative abundances of *Veillonellaceae* have been reported in the rumen of low methane emitting cattle (Wallace et al., 2015). Linseed oil suppressed the relative abundance of *Erysipelotrichaceae*, a family commonly found in the rumen of sheep. These results suggest that the lower levels of methane production associated with diets supplemented with linseed oil recorded in previous studies may be related to the selective promotion and suppression of specific bacterial families.

Although archaeal community structure was significantly different in lambs fed linseed oil (L) and the control group, the same two methanogens, *Methanobrevibacter* and *Methanosphaera* dominated both communities, albeit their relative abundance differed between treatments, with *Methanosphaera* relatively more abundant in the rumen of the L group. These two genera differ in their substrate specificity; *Methanosphaera*, spp., use CO_2_, H_2_ and formate, while *Methanobrevibacter* spp., use CO_2_, H_2_ and methanol (Liu and Whitman, 2008; Zhou et al., 2010). Thus differences in their relative abundance may reflect changes in substrate availability. There may also have been significant differences at species level that were not detected in the current study as certain substrates have been reported to favor certain species of methanogens which often have a narrow range of substrates (Danielsson et al., 2014; Henderson et al., 2015). Additionally, species-level differences in methanogenesis have been previously reported (Danielsson et al., 2017). Although methanogens are the sole producers of methane in the rumen, they are dependent on substrates, namely H_2_ and CO_2_, provided by bacterial and protozoal species to proliferate and produce CH_4_ (Hook et al., 2010). Thus, changes in other rumen microbial communities will impact on methanogens. Indeed, acetate and hydrogen producing bacteria have been linked with higher methanogen abundance in the rumen, while propionate and succinate producers are correlated with lower methanogen abundance (Van Nevel and Demeyer, 1996; Knapp et al., 2014).

Volatile fatty acid concentrations were similar in the control and linseed fed lambs despite these significant changes in microbial community structure. Like other microbiomes, the rumen microbiome contains a high level of redundancy (Weimer, 2015) which could explain why differences in bacterial and archaeal diversity and bacterial VFA correlation profiles did not impact VFA concentrations/profile.

### Effect of Linseed Oil during the Pre-weaning Phase Only

Lambs in the L-P group received linseed oil during the pre-weaning stage only and reverted to the control diet after weaning. Despite being on the same diet for approximately 10 weeks prior to sampling, the rumen bacterial community structure of the lambs in the L-P group differed significantly from those that never received any linseed oil (control group) and thus did not revert back to a structure similar to the control group once supplementation ceased. This is an interesting result, as manipulation of rumen microbiomes in later life typically only last days to weeks and this has been attributed to the redundancy and resilience present in the rumen microbiome (Hart et al., 2008; Weimer, 2015). There have been other reports of pronounced and long-lasting effects of early life dietary experiences (Distel et al., 1994; Yáñez-Ruiz et al., 2015). Both Yáñez-Ruiz et al. (2010) and Abecia et al. (2014b) reported persistently altered microbiomes and possible early-life microbial programming with diets supplemented with BMC. Primary colonizers of the gut have been shown to influence sequential colonizers (Chaucheyras-Durand and Fonty, 2002; Danzeisen et al., 2013) and thus early life diet may exert an effect by influencing primary colonizers which are important in determining the mature microbiome composition.

Even when the lambs only received linseed oil during the pre-weaning phase, most of the dissimilarity observed in bacterial community structure compared to the control group was once again due to the *Succinivibrionaceae*, which were relatively more abundant in the L-P group. Likewise, the *Veillonellaceae* were relatively more abundant in the L-P group compared to the control group whereas the *Ruminococcaceae* family were relatively more abundant in the control lambs.

*Spirochaetaceae*, a bacterial family commonly found in the rumen (Bekele et al., 2011) had a higher relative abundance in the control group than in the animals receiving the supplement during the pre-weaning period. *Fibrobacter* and *Sharpea*, found in higher abundance in low CH_4_ emitters by Kittelmann et al. (2014) were found to be relatively more abundant in the control group in the current study than in either group of lambs receiving the supplemented diet pre-weaning or throughout. Overall these results may indicate that the linseed fed lambs had a microbiome more likely to be associated with reduced methane emissions.

*Bifidobacteriaceae*, reported to confer numerous health benefits on a host including regulation of intestinal microbial homeostasis, inhibition of pathogens, modulation of local or systemic immune responses, production of vitamins and bioconversion of dietary components to bioactive compounds (Mayo and Van Sinderen, 2010) were present in very low relative abundance (0.06%) in bacterial communities in control lambs compared to both lamb groups that received linseed oil, even those that received it pre-weaning only. This may be significant for the long-term health of the linseed oil fed groups.

Once again, despite the impact of linseed oil on microbial community structure and differences in the VFA correlation profiles, no difference was observed in the VFA profiles of the L-P and control lambs, again suggesting that overall fermentation was not impacted. These results suggest that dietary treatment early in life can cause alterations in the microbiome which persist even after the diet has been changed, suggesting that a window of opportunity may exist when microbial communities could be manipulated to facilitate improved health and/or production characteristics. However, the differences observed in the microbial composition of lambs receiving the linseed supplement pre-weaning only and those that received it throughout suggest that the effects of supplementation on the microbiome may be less predictable than desired.

### Effects of Short-Term versus Long-Term Supplementation

Although rumen archaeal community structure was similar in lambs fed linseed oil throughout the study (L) and those fed the supplement pre-weaning only (L-P), significant differences were observed in bacterial community structures between these groups. This highlights the difficulty in predicting outcomes of interventions in such complex microbial networks. One possible explanation for the differing bacterial community profiles between the two groups is the effect of a second disturbance, in the form of a dietary change during a crucial milestone (weaning), on microbial communities. Multiple stressors can combine in different ways to give an overall effect which may be equal to the sum of the stressors (additive), equal to the effect of the most detrimental impact (simple comparative) or greater (synergic) or less than (antagonistic) the sum of individual disturbances. This makes the effect of multiple disturbances difficult to predict (Folt et al., 1999; O’Gorman et al., 2012; Vye et al., 2015). It is possible that introduction of an additional disturbance during early life microbial colonization of the rumen led to greater stress on the microorganisms and therefore an overall greater effect on microbial community structure. If dietary treatment had been continued through weaning (for an extra 2 weeks) the microbial profiles of the lambs fed linseed oil throughout the study and the lambs fed linseed oil short-term may have been more similar.

Although not significant, there did appear to be a trend of higher propionate and lower butyrate levels in lambs in the L-P group compared to those fed linseed oil throughout (L group). This may be relevant in the long-term for methane production. High butyrate concentrations in the rumen have been positively correlated with high methane emissions, while concentrations of propionate have been negatively correlated with methane emissions (Hungate, 1996; Lana et al., 1998; Russel, 1998; Mohammed et al., 2011). The fact that the relative abundance of three of the dominant bacteria (*Prevotella, Succinivibrio*, and unclassified *Veillonellaceae*) was negatively correlated with propionate concentration in lambs in the L group, but positively correlated with this VFA in the lambs fed the diet during pre-weaning only, suggests that either different processes were occurring in the two treatment groups or different bacteria were mediating these processes. It would be interesting to compare methane emission levels from lambs fed linseed oil short-term to those that continued to receive linseed oil for the entire study to see whether the microbial alterations achieved through short-term supplementation translated into persistent decreased methane emissions once supplementation had ceased.

## Conclusion

In conclusion, supplementation of lambs’ diets with linseed oil had a significant effect on both bacterial and archaeal community structure in the rumen. Furthermore, early life, short-term supplementation of lambs’ diets with linseed oil led to long-term changes in rumen bacterial community structure. This data highlights the importance of early life diet in shaping the composition of the mature microbiome. However, microbial community structure in the rumen of lambs fed linseed oil short-term and lambs fed linseed oil throughout the study differed, indicating that the consequences of alterations and disturbances in the rumen as the microbiome is being established in early life is difficult to predict. This study provides insight into the effect of early life dietary intervention on the ruminal microbiome and may aid in the design of strategies to improve feed efficiency, improve host health or decrease methane emissions through manipulation of the microbiome.

## Author Contributions

TL is the Ph.D. student who undertook all the laboratory work on the microbiome. TB is the PI on the animal studies and set up the experiment. ED is the PI on the microbiome studies and contributed to the design, data analysis and writing of the paper. SS is a post-doctoral fellow in the microbiology lab and was involved in all aspects of the microbiology but particularly the statistical analysis.

## Conflict of Interest Statement

The authors declare that the research was conducted in the absence of any commercial or financial relationships that could be construed as a potential conflict of interest.

## Footnotes

1 arb-silva.de/silva-license-information

## Supplementary Material

FIGURE S1 Average daily feed intake, and weight gain (ADG), from birth and weaning, for lambs on a control diet (C), lambs fed linseed oil for 16 weeks (L), and lambs fed linseed oil pre-weaning only (L-P).

TABLE S1 Complete list of phyla assigned to sequences generated through sequencing DNA extracted from rumen samples from lambs in the control group (untreated).
