# Supplementary figures and images for: Linseed Oil Supplementation of Lambs’ Diet in Early Life Leads to Persistent Changes in Rumen Microbiome Structure
Source: Front Microbiol. 2017 Aug 29;8:1656. doi: 10.3389/fmicb.2017.01656 (PMC5583589; doi:10.3389/fmicb.2017.01656)

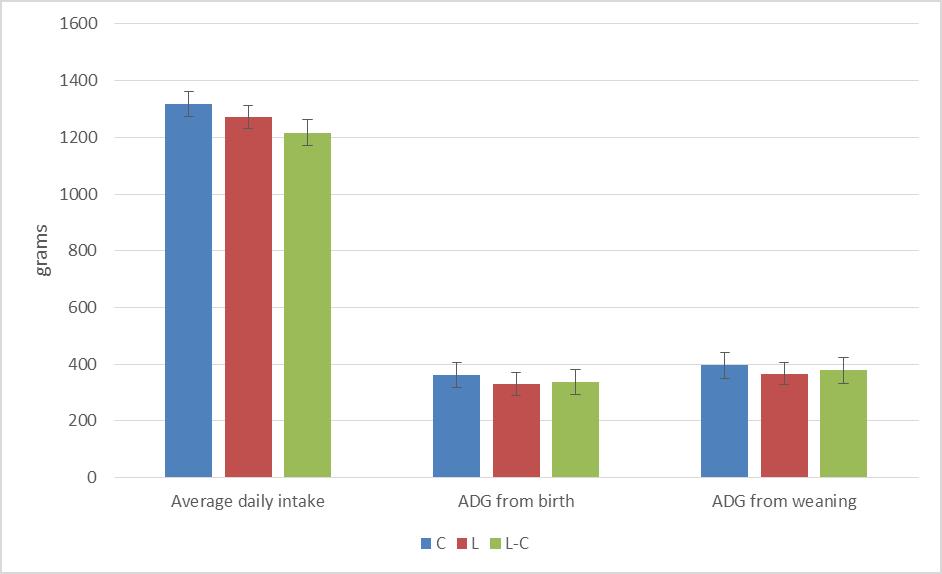

Supplement: FIGURE S1 — Average daily feed intake, and weight gain (ADG), from birth and weaning, for lambs on a control diet (C), lambs fed linseed oil for 16 weeks (L), and lambs fed linseed oil pre-weaning only (L-P). [file Image_1.jpg]
