# Supplementary material for: Linseed Oil Supplementation of Lambs’ Diet in Early Life Leads to Persistent Changes in Rumen Microbiome Structure
Source: Front Microbiol. 2017 Aug 29;8:1656. doi: 10.3389/fmicb.2017.01656 (PMC5583589; doi:10.3389/fmicb.2017.01656)
Supplement: TABLE S1 — Complete list of phyla assigned to sequences generated through sequencing DNA extracted from rumen samples from lambs in the control group (untreated). [file Table_1.PDF]

Complete list of sequences assigned to phyla based on DNA extracted from rumen samples from lambs on a control diet (C), lambs fed linseed oil for 16 weeks (L) and lambs fed linseed oil pre-weaning only (L-P)

phylum	**Control group**											
	C14	C21	C35	C22	C19	C54	C55	C25	C42	L33	L41	L48
Crenarchaeota	0	0	0	0	0	0	0	1	0	1	0	0
Euryarchaeota	1094	1112	179	118	3831	448	293	436	112	1089	463	575
Acidobacteria	5	2	0	4	6	5	6	5	1	7	2	3
Actinobacteria	328	418	90	36	2468	268	66	150	149	169	106	117
Armatimonadetes	0	0	0	1	0	0	0	0	0	0	0	0
Bacteroidetes	8937	18112	3039	1628	18267	13197	2922	8919	7359	7419	2015	7148
Chlamydiae	0	0	0	1	0	0	0	0	0	0	0	0
Chloroflexi	2	0	6	1	2	0	0	0	0	0	0	0
Deinococcus-Thermus	0	0	0	0	0	0	0	0	0	0	0	0
Fibrobacteres	59	164	1	7	78	77	11	34	9	2	14	1
Fusobacteria	1	0	0	0	0	0	0	0	0	0	0	2
Gemmatimonadetes	0	1	0	1	0	0	0	0	0	0	0	0
Nitrospira	0	0	0	0	0	0	0	1	0	0	0	0
Planctomycetes	1	1	0	1	0	0	2	0	1	1	0	1
Proteobacteria	4947	19064	2054	1060	816	943	294	906	4687	1434	329	8037
Spirochaetes	777	189	2	9	209	1	7	33	11	3	0	6
Synergistetes	33	2	8	0	9	3	3	7	28	4	1	25
Tenericutes	0	0	0	0	4	0	0	0	0	0	0	0
Verrucomicrobia	3	2	0	4	0	4	5	0	0	1	0	0
Cyanobacteria_Chloroplast	1	0	0	0	8	4	1	0	0	3	0	1
Firmicutes	9281	11165	1086	681	28675	5029	5130	8379	2227	6306	2332	4642
SR1	0	0	0	0	0	0	0	0	0	0	0	0
TM7	0	0	0	0	0	0	0	0	0	0	0	0
unclassified Bacteria	1173	2810	83	108	1460	253	176	422	193	413	97	269

Complete list of sequences assigned to phyla based on DNA extracted from rumen samples from lambs on a control diet (C), lambs fed linseed oil for 16 weeks (L) and lambs fed linseed oil pre-weaning only (L-P)

**L group**						**L-P group**								
L43	L20	L30	L51	L32	L38	LC40	LC29	LC45	LC46	LC31	LC52	LC49	LC23	LC17
0	0	1	1	0	0	0	1	0	1	0	0	0	0	0
433	348	1167	485	212	182	1151	4007	24	44	980	1863	1532	2462	2205
5	1	4	1	2	3	3	8	7	2	3	6	1	4	9
71	77	146	102	109	188	353	2170	4	9	1022	747	474	1467	1856
0	0	0	0	0	0	0	0	0	0	0	0	0	1	0
5080	4172	8037	6584	5923	3222	12404	49355	288	960	16459	9137	6186	12349	13700
0	0	0	0	0	0	0	0	0	0	0	0	0	0	0
1	0	1	0	0	0	4	11	0	1	0	1	1	0	3
0	0	0	0	0	0	0	0	0	0	0	0	0	0	0
11	0	36	3	1	1	3	80	1	0	222	103	76	261	0
0	0	0	0	0	0	0	1	0	0	1	0	0	0	0
0	0	0	0	0	1	0	0	0	0	0	0	1	0	0
0	0	0	0	0	0	0	0	0	0	0	0	0	0	0
1	2	0	1	1	1	1	2	5	1	0	1	3	0	2
4402	5696	1218	5422	4470	4026	7866	49979	171	932	7792	4274	4912	9532	5851
41	8	9	23	6	1	22	44	0	0	99	628	653	20	16
4	20	15	8	27	9	10	98	0	0	55	27	23	11	0
0	0	0	0	0	0	0	0	0	0	0	0	0	0	0
1	1	0	0	1	2	3	1	2	0	17	4	2	2	2
0	0	4	0	0	0	2	12	0	0	56	92	3	4	0
5082	2883	6347	3721	6562	3373	7031	39884	199	1081	11037	8807	6895	13363	18866
0	0	0	0	0	0	13	39	0	0	0	0	0	0	0
0	0	0	0	0	0	0	6	0	0	0	0	1	5	0
47	148	757	328	108	62	146	2839	22	12	1278	1442	987	2729	656
